# Mixed Reality (Holography)-Guided Minimally Invasive Cardiac Surgery—A Novel Comparative Feasibility Study †

**DOI:** 10.3390/jcdd12020049

**Published:** 2025-01-27

**Authors:** Winn Maung Maung Aye, Laszlo Kiraly, Senthil S. Kumar, Ayyadarshan Kasivishvanaath, Yujia Gao, Theodoros Kofidis

**Affiliations:** 1 Department of Cardiac, Thoracic and Vascular Surgery, Division of Congenital Cardiac Surgery, National University Hospital, National University Health System, Singapore 119228, Singapore; kiraly_laszlo@nuhs.edu.sg (L.K.); senthil_kumar_subbian@nuhs.edu.sg (S.S.K.); surtk@nus.edu.sg (T.K.); 2 Yong Loo Lin School of Medicine, National University of Singapore, Singapore 117597, Singapore; ayyadarshan.kasivishvanaath@mohh.com.sg; 3 Department of Surgery, National University Hospital, National University Health System, Singapore 119228, Singapore; yujiagao@nuhs.edu.sg; 4 Department of Public Health, Semmelweis University, 1085 Budapest, Hungary

**Keywords:** minimally invasive cardiac surgery, mixed reality, virtual reality, augmented reality, 3-dimensional holographic image, HoloLens^®^ 2

## Abstract

The operative field and exposure in minimally invasive cardiac surgery (MICS) are limited. Meticulous preoperative planning and intraoperative visualization are crucial. We present our initial experience with HoloLens^®^ 2 as an intraoperative guide during MICS procedures: aortic valve replacement (AVR) via right anterior small thoracotomy, coronary artery bypass graft surgery (CABG) via left anterior small thoracotomy (LAST), and pulmonary valve replacement (PVR) via LAST. Three-dimensional (3D) segmentations were performed using the patient’s computer tomography (CT) data subsequently rendered into a 3D hologram on the HoloLens^®^ 2. The holographic image was then superimposed on the patient lying on the operating table, using the xiphoid and the clavicle as landmarks, and was used as a real-time anatomical image guide for the surgery. The incision site marking made using HoloLens^®^ 2 differed by one intercostal space from the marking made using a conventional surgeon’s mental reconstructed image from the patient’s preoperative imaging and was found to be a more appropriate site of entry into the chest for the structure of interest. The transparent visor of the HoloLens^®^ 2 provided unobstructed views of the operating field. A mixed reality (MR) device could contribute to preoperative surgical planning and intraoperative real-time image guidance, which facilitates the understanding of anatomical relationships. MR has the potential to improve surgical precision, decrease risk, and enhance patient safety.

## 1. Introduction

Unlike the abdominal wall, the chest wall is rigid and does not allow changes in the visual angle. The path of access to the three-dimensional (3D) target structures is deep, and the angle is fixed and restricted [1]. Appropriate incision and exposure to allow adequate visibility and access are of paramount importance in MICS [1,2]. Meticulous preoperative planning, especially determining the site of entry into the chest, and real-time intraoperative image guidance are necessary for successful surgery [3,4]. The main aim of the minimal invasive surgery (MIS) approach is to reduce collateral tissue damage and morbidity without risking the completeness of the intended surgical repair [1,2]. Possible advantages of MIS include faster and better postoperative mobilization, independence, fewer infections, less blood loss and need for transfusion, less trauma, less re-exploration, less postoperative lactate, and fewer arrhythmias [2]. Current preoperative imaging, e.g., 2/3D echocardiography, computer tomography angiography (CTA), and magnetic resonance imaging (MRI), display 2D or reconstructed 3D images, typically on a flat 2D screen, requiring the surgeon to create a mental model and apply it onto the patient’s real anatomy during surgical planning and/or in surgery [1,5]. As spatial intelligence is variable, it may be difficult for individual surgeons to establish the 3D spatial relationship and correct depth perception of the patient’s anatomy using preoperative imaging and their mental model [3].

**Figure 1:**
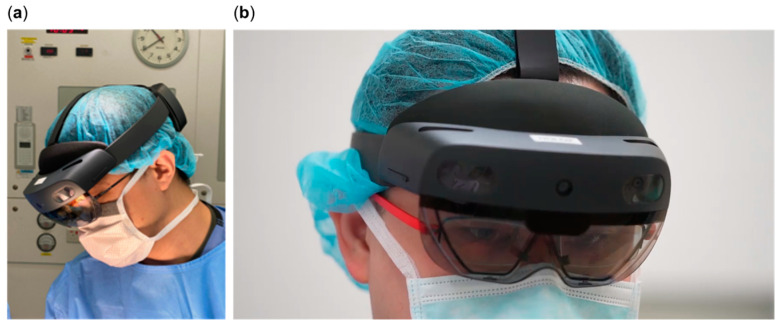
Surgeon wearing HoloLens^®^ 2: (**a**) Looking at the patient on operating table; (**b**) looking through the clear visor onto the hologram.

**Figure 2:**
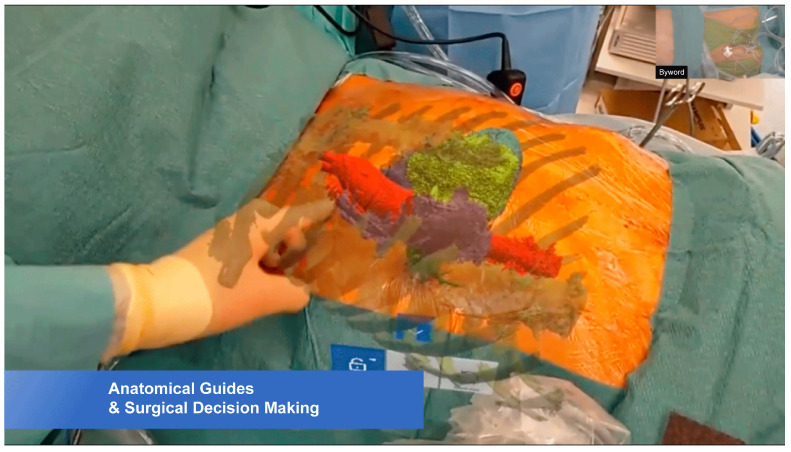
HoloLens^®^ 2-guided minimally invasive surgery for aortic valve replacement.

This cognitive problem can be overcome by using HoloLens^®^ 2 (Microsoft Corporation, Redmond, WA, USA), a head-mounted mixed-reality (MR) holographic device [6,7,8] (Figure 1a,b). It provides an immersive experience by superimposing a holographic 3D-rendered model onto the patient’s anatomy [3,9] (Figure 2). Furthermore, the surgeon’s line-in-sight is maintained throughout the procedure so that the operator is not distracted by another screen [9,10].

In virtual reality (VR), the real environment is replaced by a virtual one, whereas augmented reality (AR) overlays virtual objects in the real, physical environment without the user being able to interact with them. Pokémon Go is a prime example of AR [4]. Mixed reality (MR) combines VR and AR [3,4,10,11], allowing surgeons to have simultaneous interactions with the virtual and physical world by superimposing holographic 3D images onto the operative field; thus, MR increases the user’s experience of depth and perception [4,10].

Using HoloLens^®^ 2, the patient’s own virtual holographic model of the heart is anchored by selected landmarks on the thoracic wall, and the model is projected inside the chest cavity [4]. The main advantage of holograms placed inside the operative field is that surgeons have a better appreciation of the vector and magnitude of movement required [9]. HoloLens^®^ 2 guides the surgeon to locate the most appropriate site of incision and auxiliary ports so that optimal visibility of and access to the operative field can be achieved [3].

To date, there are no reports in the MICS literature describing the use of HoloLens^®^ 2 for improving surgical access. We present—to the best of our knowledge—a novel comparative study of three MICS procedures using HoloLens^®^ 2 for preoperative planning and for real-time intraoperative guidance [9].

## 2. Materials and Methods

### 2.1. Computed Tomography Angiography (CTA)

A third-generation dual-source 2 × 192-slice scanner (SOMATOM Force, Siemens Healthcare, Forchheim, Germany), and the images are obtained using a prospectively ECG-triggered high-pitch spiral mode (Turbo Flash, 2 × 192 × 0.6 mm detector collimation, Siemens Healthcare) [9]. The slice thickness is based on detector collimation, which is 0.6 mm. The CT protocol was designed by radiologists and radiographers in consultation with the equipment vendor.

The images were then uploaded onto the HoloLens^®^ 2 system to be rendered into a 3D holographic model [6].

### 2.2. Hologram Creation

Consent for photography or videography was obtained from each patient in accordance with institutional guidelines. CTA DICOM data were extracted from CT scans. Segmentation and alignment of the images were performed. These images were exported as a DICOM standard dataset, which was then uploaded onto HoloLens^®^ 2 via Virtual Surgery Intelligence software version 1.5 (apoQlar GmbH, Hamburg, Germany) that generated the reference cardiothoracic hologram [9]. Additionally, segmentation of individual structures within the raw scan images was performed using 3D Slicer (The Slicer Community, www.slicer.org, v4.11.20210226) via manual segmentation to identify the individual cardiac chambers, valves, coronary arteries, and internal mammary arteries. The segmented images were exported as standard tessellation language (STL) files onto virtual surgery intelligence (VSI) and used for image superimposition and visualization [9]. The entire process of hologram creation took approximately 3 h. It was reviewed by the surgical team preoperatively.

### 2.3. Surgical Procedures

The patient was positioned on the operating table, and the surgeon marked the preferred site of entry employing conventional anatomical landmarks and their mental model. Then, they superimposed the reconstructed patient’s cardiac hologram onto the chest using the xiphoid and the clavicle as reference landmarks for a real-time anatomical assessment. This enabled the surgeon to look “into” the chest organ topography in their line-in-sight [10]. The best incision site was again marked. In addition, insertion points of surgical instruments and accessories were chosen in reference to the intended incision site marked using HoloLens^®^ 2. Assessment with the hologram also enabled anticipation of difficulties and to foresee any possible spatial confinement.

The technicalities of superimposing the 3D-rendered image on the operative field were initially challenging. They required the surgeon to virtually hold the holographic image in mid-air and place it onto the patient’s chest using the landmarks as accurately as possible [3,10]. Surgeons had preoperative training sessions of approximately 20 min and quickly familiarized themselves with the movements of the holographic imagery [10].

The first procedure was an MIS aortic valve replacement (AVR) via right anterior small thoracotomy using a totally endoscopic technique (Figure 2). Using HoloLens^®^ 2, a holographic image was superimposed onto the actual patient’s chest. The position of the aorta and aortic valve annulus were visualized. With this guidance, the incision was made, and the chest was entered via the second intercostal space. The AVR was performed in the usual manner.

The second procedure was an MIS off-pump coronary artery bypass graft surgery (CABG) via left anterior small thoracotomy (LAST). A similar process was performed. The fixed holographic 3D image guided the best way for the surgeon to access the most appropriate intercostal space and to plan the entry into the chest cavity. It also facilitated decision-making over the placement of bypass grafts and projected the degree of difficulties of exposure.

**Figure 3:**
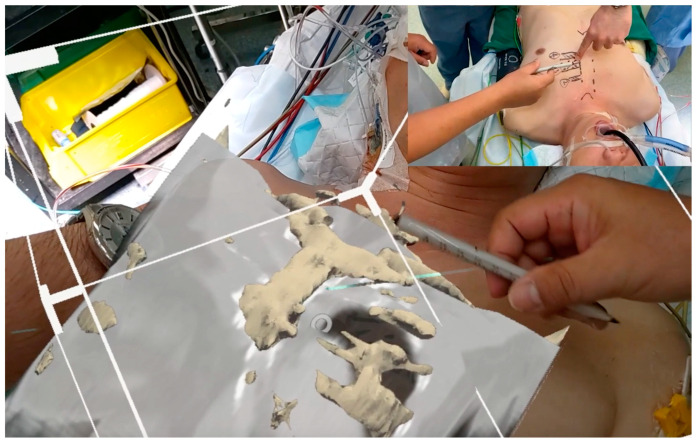
HoloLens^®^ 2 guided minimally invasive surgery for pulmonary valve replacement.

The 3rd case was an MIS pulmonary valve replacement (PVR) (Figure 3). PVR following repair of Tetralogy of Fallot in childhood is conventionally carried out via redo-median sternotomy. In order to avoid redo-median sternotomy, PVR was planned via LAST. Three-dimensional superimposed holographic visualization adequately projected the position of the main pulmonary artery (MPA) and pulmonary valve annulus (PVA) and guided the most appropriate intercostal space to enter the chest.

## 3. Results

Approximately 3 hours of preoperative worktime was required to create a 3D holographic image to be viewed on the device. Production was performed by a different in-house team.

The xiphoid process and the clavicle provided consistent reference points to lock the hologram on the patient’s chest, ensuring accurate placement. Hologram superimposition and fine adjustments were straightforward as surgeons had had prior training and were familiar with the technicalities. The OR time taken for the process was less than 10 min from mounting the device on the surgeon’s head to marking the incision site. Dictated by the structures of interest, the most accurate and smallest, incision site could be selected.

In all three cases, the incision site marked with and without the HoloLens^®^ 2 differed by one intercostal space. HoloLens^®^ 2 provided more appropriate access to the operating field: one intercostal space cranial in MIS AVR and MIS PVR and one intercostal space caudal in MIS CABG. The endoscopic tool distribution (camera insertion, left ventricular vent, and their relation to the structures of interest/chest wall) could be better appreciated and/or planned. HoloLens^®^ 2 references were found to be more suitable than the markings based on the surgeon’s interpretation of the structures of interest from preoperative imaging. The transparent visor of the HoloLens^®^ 2 allowed unobstructed views of the operating field. The operative time in 3 MIS cardiac surgical procedures described, with the use of HoloLens 2, was reduced by a mean duration of 34.3 ± 3.2 min. This could be attributed to the entry site being the most accurate without requiring change during the procedure, which can be time-consuming. A survey was also conducted to determine the level of user acceptability for MR devices in surgery. This survey was conducted in 2022, with 30 surgeons who had trialed the solution. Survey feedback was collected using a validated User Evaluation Questionnaire (UEQ), that uses a 26 point matrix to compare the new solution with existing devices. The survey results showed overwhelming positive response to the use of MR in surgery, as shown in Suppelementary File S2.

In this work, the study presented in [12] is expanded upon.

## 4. Discussion

The concept of holographic visualization and the necessary technology have existed for many decades [13,14]. The use of this technology in the medical field has been delayed by the bulky nature of the devices [3,13] and the inability to view high-quality images [3]. Microsoft HoloLens^®^ 2, a head-mounted MR holographic device, was first made commercially available in 2016 [3] and is currently widely used in various medical and surgical subspecialties. MR technology, interconnected with both VR and AR, allows the user to interact with virtual reality in reality. MR has been applied in numerous medical [15,16,17,18,19,20] and surgical specialties, e.g., orthopedic surgery [21], neurosurgery [22], urology [23], general surgery [24], etc. Advantages have been demonstrated in both educational and clinical settings to help medical professionals better understand complex anatomical structures, so the modality is available for preoperative surgical planning and intraoperative guidance [25,26,27]. VR and 3D-printed heart models were also found to be superior for diagnostic assessment, medical education, and preoperative planning in comparison with conventional visualization techniques [28].

### 4.1. Benefits

MR devices offer multiple presumed benefits in MICS intraoperative visualization [10,29,30]. The patient’s own 3D holographic model superimposed on the actual anatomy ascertains spatial anatomy. The hologram can be adjusted and rendered to anatomical landmarks in real time [9]. All this contributes to incisional precision and accuracy of access to the target structures [31,32]. In fact, in each of our presented cases, HoloLens^®^ 2’s superimposed model recommended a different intercostal space for chest entry from the one assumed by the surgeon’s mental model. Modification of chest entry by one intercostal space can achieve a significantly better visualization of the structures of interest in a confined operating space in minimally invasive cardiac surgery. The superiority of HoloLens^®^ 2 can be attributed to the fact that holograms not only revealed the appropriate depth and relationships but also the angulation and vector orientation of optimal access.

Furthermore, holographic models propose an optimal distribution and orientation of auxiliary ports for manipulation and visualization. Intrathoracic spatial anatomical findings proved the holographic recommendations and no need for interspace and/or port adjustment occurred. We propose that intraoperative MR technology is the ultimate step in the meticulous preoperative planning process that is required for MICS.

HoloLens^®^ 2’s superimposed model, in line with the surgeon’s vision, optimizes ergonomics, i.e., the surgeon can focus on the progress of the procedure without any interruption to consult with an external 2D flat screen [10] and/or disturbance of the visual–motor axis or hand–eye coordination [7,33]. Assumed benefits include improved surgical performance, reduced procedural length, more accurate visualization, and identification of anatomical structures leading to decreased risk of iatrogenic injuries [5,10,30]. The operator’s integrated perception of virtual and real structures and their spatial relationship contributes to cognitive coherence [11], reduced operator stress and fatigue, and less risk of complications; all this potentially translates into improved patient safety. Intraoperative holography, not involving physical contact with any material structure, is a non-invasive, aseptic modality [29].

MR offers a competitive advantage in avoiding median resternotomy in selected cases. Redo-sternotomy carries inherent risks of potentially fatal injury of the mediastinal structures upon chest entry [34,35]. CT angiography has been advocated as part of the preoperative work-up [36]. CT datasets converted into holographic models open an avenue for an alternative approach, e.g., LAST for PVR [37]. Holographic incisional guidance provides direct access to the target structures and significantly reduces operative risks while increasing precision [38]. The general acceptance and initial user experience were positive throughout our study.

### 4.2. Limitations

The alignment of 3D holographic images onto a patient’s chest was carried out manually using only two anatomic landmarks, namely the xiphoid and clavicles. More alignment points on exposed landmarks might be required for better precision [4]. Manual alignment needs to be mastered and is time-consuming [10]. Similarly, accommodation to HoloLens^®^ 2 headgear requires some habituation. Co-application of regular head-mounted surgical equipment, such as surgical loupes and headlights typically worn by cardiac surgeons during surgery might be difficult or even impossible with HoloLens^®^ 2 [4]. The holographic images are static [4], and no magnification has yet been integrated into the models [10]. HoloLens^®^ 2 was only applied in the initial phases of the operations, i.e., during chest entry and intrathoracic orientation; the surgical procedures did not require additional spatial guidance. Our study of three surgical procedures only represents our initial experience with MR and HoloLens^®^ 2. The modality requires more exposure with a wider spectrum of anatomies and complexities in order to identify clinical applicability, accuracy, user comfort, and the device’s place in the MICS armamentarium.

## 5. Conclusions

The use of an MR device in MICS could improve our understanding of anatomical spatial relationships. As an intraoperative form of real-time image guidance, it may be regarded as an important last step in pre- and intraoperative planning that offers improved precision of access and better intrathoracic orientation. In-line 3D visibility conjoining the patient’s intrathoracic anatomy with the superimposed in-line reconstructed holographic model optimizes the surgeon’s ergonomics and potentially enhances patient safety. The real value and place of the modality need further evaluation in an extended cohort of patients with a wider surgical spectrum and more degrees of complexity.

## Data Availability

The original contributions presented in this study are included in the article/Supplementary Materials. Further inquiries can be directed to the corresponding author.

## Supplementary Materials

The following supporting information can be downloaded at https://www.mdpi.com/article/10.3390/jcdd12020049/s1, File S1: HoloLens 2 Video; (https://youtu.be/rlC2IilA4i8, accessed on 11 September 2024); File S2: Survey for use of HoloLens 2 in MIS Cardiac Surgery; File S3: Manual Segmentation Protocol.
