# Supplementary material for: Mixed Reality (Holography)-Guided Minimally Invasive Cardiac Surgery—A Novel Comparative Feasibility Study
Source: J Cardiovasc Dev Dis. 2025 Jan 27;12(2):49. doi: 10.3390/jcdd12020049 (PMC11856421; doi:10.3390/jcdd12020049)
Supplement: Supplementary file 1 [file jcdd-12-00049-s001.zip › File S2 - Survey for use of HoloLens 2 in MIS Cardiac Surgery.pdf]

<u>Survey on the usefulness of the HoloLens 2 (Microsoft), Mixed Reality Device, for Cardiac Surgery</u>

Please do not mention your name to maintain anonymity.

1. **Is the concept of use of HoloLens 2 (Microsoft), Mixed Reality device, suitable in the surgery that you are performing especially in Minimally Invasive Surgical procedures?**
   A. Might be suitable
   B. Quite likely to be suitable
   C. Extremely likely to be suitable

2. **At which phase of the surgical procedure, you would find it most useful/practical/functional/helpful to incorporate HoloLens 2 to the surgery that you are performing? (*Please tick more than 1 answer if applicable)***
   A. Pre-operative viewing of the anatomical structure of interest
   B. In pre-operative planning stage
   C. As an intra-operative guidance during surgical procedure.
   D. As an educational tool
   E. All the above

3. **Is it easy to learn to use HoloLens 2 (Manipulating the Hologram, Positioning and Fixating the Reconstructed 3-Dimensional Holographic model of structure of interest onto real patient, use it for intra-operative guidance)?**
   A. Difficult to learn and not user friendly
   B. Take some time to get used to the technique
   C. Very easy to learn to use

4. **Approximately how long did it take you to learn to use and be proficient in using HoloLens 2?** (Please tick)
   A. 10 - 15 Minutes
   B. 15 - 30 Minutes
   C. 30 - 45 Minutes
   D. 45 - 60 Minutes

5. **How do you find the image quality of 3-Demensional Holographic model of the structure of interest in HoloLens 2 compared to reconstructed 3-Demensional images from MRI or CT scans?**
   A. Not similar at all, lacks important details
   B. Somewhat similar but some minor details are missing
   C. Exact replica of reconstructed 3-Dimensional images from MRI or CT scans images

6. **The weight of the HoloLens 2 is approximately 500g. Do you find it uncomfortable to wear during surgery?**

   A. No

   B. Yes

7. **Would you likely to use the HoloLens 2 during your operating procedures, especially in minimally invasive surgery?** *(Please tick)*

   A. Extremely unlikely

   B. Somewhat unlikely

   C. Neutral

   D. Somewhat likely

   E. Extremely likely

8. **On an added useful scale of 1 to 6 (1 being 'not all' and 6 being 'very useful') do you think Halogens 2 adds value to your procedures**? *(Please circle)*

   |1| |2| |3| |4| ||5 |6|

9. **Would you likely to recommend the use of HoloLens 2 to other surgeons and other surgical subspecialities?** *Please* tick)

   A. Yes

   B. No

   C. I am indifferent

Survey Responses from Surgeons			
Questions \ Procedures	MIS AVR	MIS PVR	MIS CABG
1	C	C	C
2	E	E	E
3	C	C	C
4	B	A	A
5	C	C	C
6	A	A	A
7	E	E	E
8	6	6	6
9	A	A	A
