# Supplementary material for: Mixed Reality (Holography)-Guided Minimally Invasive Cardiac Surgery—A Novel Comparative Feasibility Study
Source: J Cardiovasc Dev Dis. 2025 Jan 27;12(2):49. doi: 10.3390/jcdd12020049 (PMC11856421; doi:10.3390/jcdd12020049)
Supplement: Supplementary file 1 [file jcdd-12-00049-s001.zip › File S3 - Manual Segmentation Protocol.pdf]

Manual Segmentation Protocol

1. 0.6mm extra-fine cut images of CT scans performed on the patient is extracted from the radiology department in DICOM format, with anonymisation of patient data as per DSRB approval
2. Raw DICOM images are subsequently uploaded onto 3D Slicer
3. Segmentation is performed on the master volume using 'Grow from Seeds' methodology
4. Data imputation is performed using 'Fill between slices' for space between image slices
5. 15-20% image smoothening is performed on the final segmented image
6. segmented data is exported as STL file format before uploading onto the HoloLens 2 device using Virtual Surgery Intelligence software
